# Teachers as multipliers of knowledge about schistosomiasis: a possible approach for health education programmes

**DOI:** 10.1186/s12879-022-07829-x

**Published:** 2022-11-14

**Authors:** Felipe Leão Gomes Murta, Cristiano Lara Massara, Maria Gabriela Rodrigues, Lilian Christina Nóbrega Holsbach Beck, Tereza Cristina Favre

**Affiliations:** 1 Fundação Oswaldo Cruz, Instituto Leônidas E Maria Deane (ILMD), Manaus, Amazonas Brazil; 2 Instituto de Pesquisa Clínica Carlos Borborema. Fundação de Medicina Tropical Dr. Heitor Vieira Dourado, Manaus, Amazonas Brazil; 3 Universidade Do Estado Do Amazonas, Escola Superior de Ciências da Saúde, Manaus, Amazonas Brazil; 4 Fundação Oswaldo Cruz, Instituto René Rachou, Belo Horizonte, Minas Gerais Brazil; 5 Fundação Oswaldo Cruz, Instituto Oswaldo Cruz, Rio de Janeiro, Rio de Janeiro Brazil

**Keywords:** Health education, Schistosomiasis *mansoni*, Teacher training, Schoolchildren, Endemic area, Mobilisation, Brazil

## Abstract

**Background:**

In the past decade, Brazil has significantly reduced the prevalence of schistosomiasis through a combined effort of early treatment of infected people, expansion of basic sanitation infrastructure and educational measures. Despite these efforts, in some areas, prevalence of schistosomiasis exceeds 20% of the school population, who lack knowledge of the risks of the disease. Action can be taken in schools to empower this population about their health condition. This paper describes the role of the teacher as a multiplier of knowledge about schistosomiasis and proposes two different approaches to training these teachers.

**Methods:**

This study used mixed methods to evaluate training of teachers and educational intervention with those teachers’ pupils. Two training courses, each with 40 h of face-to-face activity, were offered to 19 teachers, using two different but complementary approaches, based on theoretical references and specific educational strategies: Critical Pedagogical Approach (Training Course I, held in 2013) and Creative Play Approach (Training Course II, held in 2014).The courses included classroom activities, laboratory and field work. After the training, the teachers conducted activities on schistosomiasis with their pupils. These activities involved constructing educational materials and cultural productions. The pupils’ knowledge about the disease was evaluated before the activities and 12 months later. The teachers’ acceptance and perceptions were assessed through structured interviews and subsequent thematic analysis. The *Shistosoma mansoni* infection status of teachers and their students was also assessed using the Kato Katz stool test.

**Results:**

The parasitological study showed 31.6% of the teachers and 21.4% of the pupils to be positive for *S. mansoni*. The teachers’ knowledge of important aspects of schistosomiasis transmission and prevention was fragmented and incorrect prior to the training. The teachers’ knowledge changed significantly after the training and they were strongly accepting of the pedagogical methods used during the training. The level of their pupils’ knowledge about the disease had increased significantly (p < 0.05). However, pupils responded that, even after the educational activities, they still had contact with the city’s contaminated waters (p > 0.05).

**Conclusions:**

The results of this study underline the importance of schools and teachers as partners in controlling and eliminating schistosomiasis. Teacher training on the disease significantly increases their pupils’ knowledge, reflecting empowerment with regard to local health conditions.

**Supplementary Information:**

The online version contains supplementary material available at 10.1186/s12879-022-07829-x.

## Background

Schistosomiasis, one of the world’s most important neglected diseases, is distributed across 78 countries in tropical and subtropical areas, where around 236.6 million people are exposed to the risk of infection by trematodes of the genus *Schistosoma* [1].

The species endemic in the Americas is *Schistosoma mansoni*, and the World Health Organisation (WHO) estimates that 25 million people live in risk areas for schistosomiasis, particularly in Brazil and Venezuela [2].

Before the 1950s, there are no statistical data showing the real prevalence of schistosomiasis in Brazil. From 1949 to 1978, only two national surveys on the prevalence of schistosomiasis in school-age children were carried out in the country [3]. Therefore, from 1970 to 2015, the main data on the burden of the disease in the country were obtained mainly from scientific research carried out in communities, specific cities, and villages [4–8]. Between 2015 and 2018, Brazil’s National Schistosomiasis Prevalence Survey conducted among schoolchildren, which estimated the prevalence of schistosomiasis at 1.0%, suggesting a significant decline since two earlier surveys conducted in the 1950s (10.0%) and 70s (6.9%) [9]. Despite efforts to combat the disease in recent years, there are still places in Brazil where prevalence continues as high as 20.0%, where basic sanitation is lacking and Human Development Index (HDI) scores are low [10, 11].

The highest prevalence and intensities of infection by *S. mansoni* and soil-transmitted helminthiasis are found among school-age children (6 to 15 years old), who are an important source of environmental contamination [12, 13]. For that reason, they are the main target for WHO recommendations and preventive chemotherapy programmes in several endemic countries [14]. Children’s adherence to diagnosis, treatment and other control measures implemented by local health teams has been demonstrated to be a key factor in the success of programmes directed to schistosomiasis and dracunculiasis [15, 16]. The various schistosomiasis control strategies can be made more successful and sustainable if they are combined with continued health education programs able to engage the target population and address specific local features of the infection [17, 18]. Introducing health education into the routine of schoolchildren living in endemic municipalities can be one way of building knowledge to encourage preventive practices and attitudes in the communities [19, 20].

In 2020, the WHO developed a roadmap for neglected tropical diseases, to be applied in the period from 2021 to 2030. It set goals and milestones for the prevention, control, and elimination or eradication of 20 diseases, among them schistosomiasis. The roadmap comprises three main pillars to support efforts to attain the goals, one of which is to identify cross-cutting approaches, particularly emphasising awareness-building in the population and community in combination with health education measures [21]. The implementation of sanitary facilities and access to safe drinking water alone does not guarantee a reduction in the prevalence of the disease in an endemic area and must be accompanied by educational actions with the target communities to be more effective [22, 23].

Education strategies that build permanent dialogue with the target population are useful tools for implementing control and prevention measures for disorders affecting the most vulnerable population groups. Social researchers note that health interventions have been most successful in changing population behaviour, even in developing countries, when they were culturally attractive, took local practices into consideration, and endeavoured to temper scientific knowledge with local knowhow, rather than limiting themselves just to projecting a message designed to be culturally appropriate [21]. The school has traditionally been used as a place for learning by formal teaching. However, many studies in Brazil and other endemic countries have shown a broader role for schools as a space for health promotion, non-formal teaching and addressing issues relating to schistosomiasis [16, 24–27].

This study evaluated whether, when applied to schistosomiasis, two educational approaches directed to primary and lower secondary school teachers and tailored to epidemiological conditions in the endemic municipality where they lived had contributed to improving knowledge of the endemic, and whether that improvement was reflected positively in the level of knowledge acquired by their pupils.

## Methods

### Study site

The study was conducted, from 2013 to 2015, in an area where schistosomiasis is endemic in the municipality of Malacacheta, Minas Gerais State, Brazil, which has 18 public schools. In 2010, the municipality had an Human Development Index (HDI) of 0.618 and a population of 18,776, 37.0% of which lived in rural areas, 17.3% in extreme poverty and 3943 (21%) were of school-age [28].

**Fig. 1:**
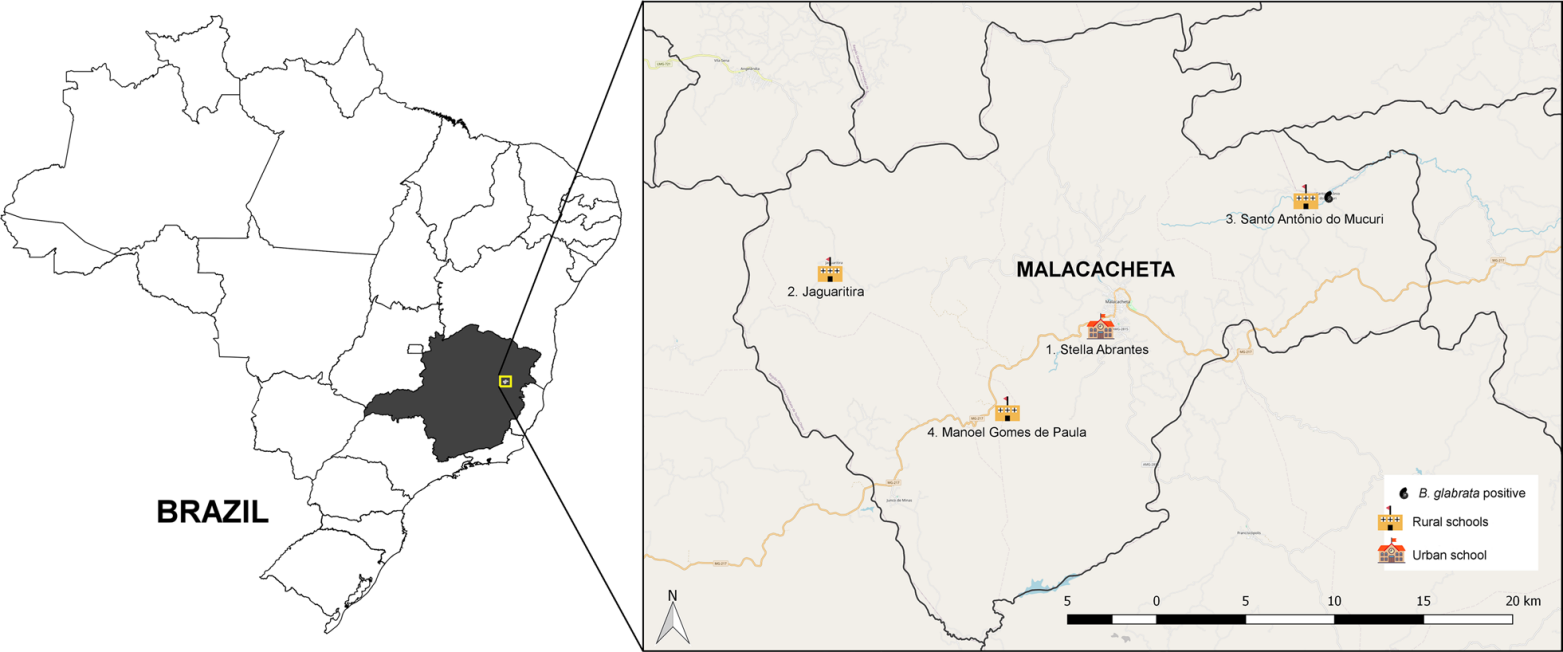
Spatial distribution of the four schools selected for the study. Obs.: showing the body of water where the specimen of *B. glabrata* naturally infected with *S. mansoni* was found. Image from authors

The baseline parasitological survey conducted by the study team among the municipality’s schoolchildren included Malacacheta’s 18 public schools and 2519 primary and lower secondary school pupils, 21.4% of whom were found to be infected with *S. mansoni* [10]. For this study, four of those schools (three rural and one urban) were chosen at random from a selection that included those with the largest numbers of pupils and the highest percentages of testing positive for *S. mansoni* (Fig. 1).

Malacological surveys in 15 of the bodies of water in the municipality, in 2013 and 2015, recorded the presence of *Biomphalaria straminea* and *B. glabrata* at various breeding sites*.* In 2013, a specimen of *B. glabrata* naturally infected with *S. mansoni* was collected close to one of the study schools (unpublished data).

### Study design and participants

This study used mixed methods with qualitative and quantitative approaches to evaluate training sessions for teachers and an educational activity with their respective pupils.

The study included 19 primary and lower secondary school teachers, five of them were men and 14 were women. They were designated by the school principals and after the presentation of the study, we selected the teachers who were interested in voluntarily participating in the research. Their backgrounds were multidisciplinary, with length of teaching experience ranging from 4 to 15 years, and they taught at the study schools. All students (n = 376) who were under the supervision of the 19 teachers included were invited to participate in the study, which characterized the target group of the educational activity conducted by the teachers. After a 24% loss of segment caused by school absenteeism and change of address, 276 pupils (148 boys and 128 girls) were included in the statistical analysis of the study. All were enrolled in the 5th and 6th years of school, were from 11 to 16 years old and 58.7% lived in the rural area of the municipality. None of the participants had an established relationship with an author prior to study commencement.

### Pedagogical approaches

Two training courses, each comprising 40 h of in-person activities, were offered to the teachers in the study. Two researchers (TF and LB) were the tutors responsible for both trainings and the educational activities including theory classes and practical laboratory and field work; that is, two different but complementary approaches were used, drawing on specific theoretical references and educational strategies: Critical Pedagogical Approach (Training Course I, held in 2013) and Creative Play Approach (Training Course II, held in 2014). Different teaching materials and educational techniques were used in each course (Additional file 1).

The central idea of the study was to empower the primary and lower secondary school teachers in the study to work in the classroom with their pupils as multipliers of knowledge about schistosomiasis. At the end of the two courses of training, a guide of activities with specific content and educational materials was assembled. The guide was applied by the teachers, with no interference from the researchers. Each of the 19 teachers participated in only one of the training courses in order to prevent bias in the analysis.

**Fig. 2:**
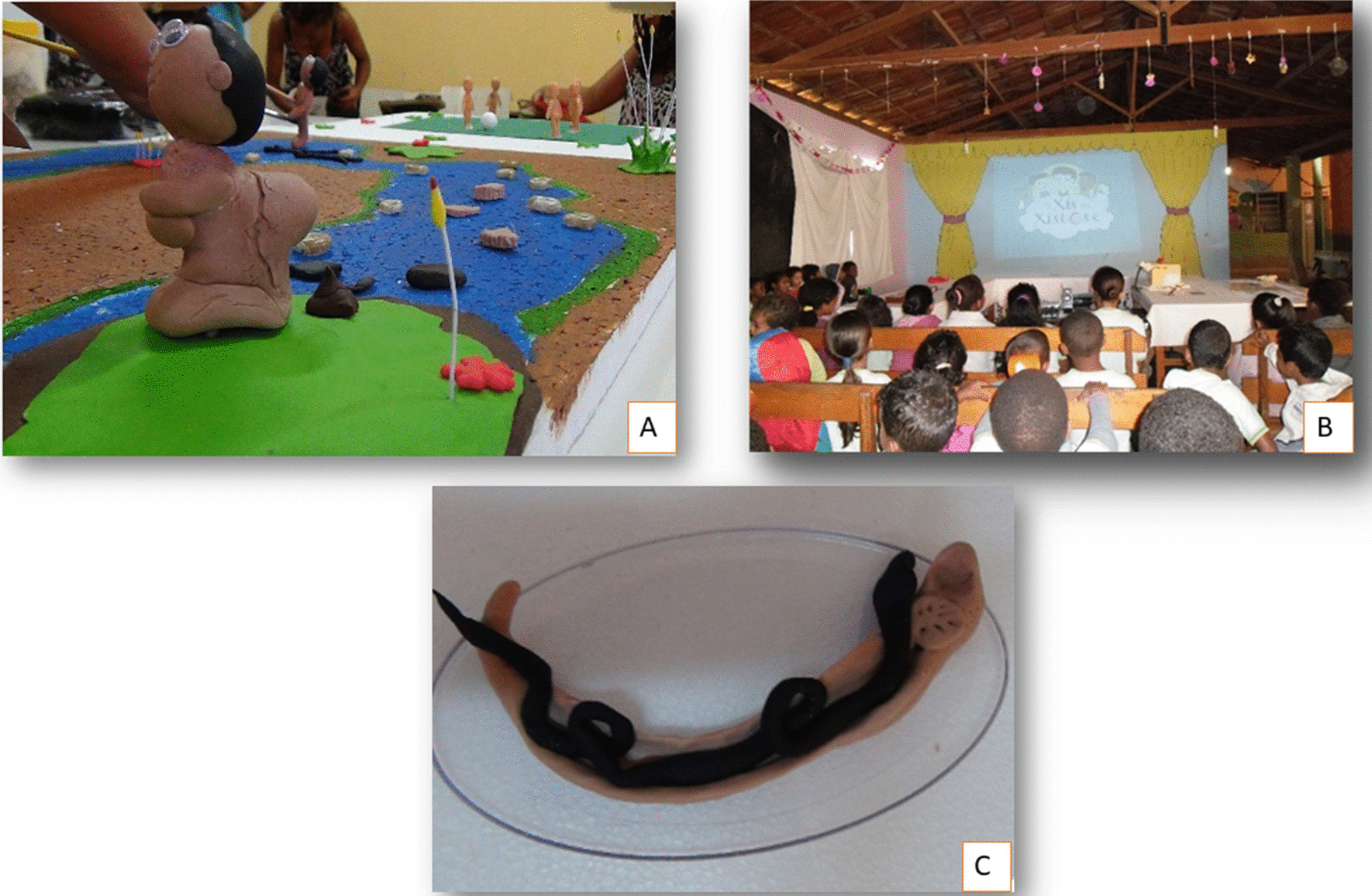
School teachers in schistosomiasis training. **A** Preparation of a model representing the risk area and the parasite cycle. *Biomphalaria* sp. shells were used (**B**) Teacher developing interactive educational materials. **C** Educational activity with students. Image from authors

**Fig. 3:**
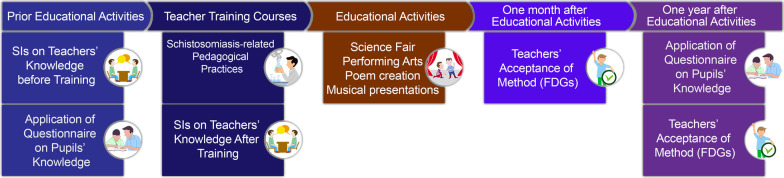
Timeline of study events. Image from authors

The teachers addressed the subject of schistosomiasis by preparing projects, which they tailored to the particular situation of each school. The projects involved developing educational materials and cultural productions, such as plays, texts (poetry and prose) and music. These productions were put on at thematic fairs at each school (Fig. 2). The pupils’ knowledge about the disease was assessed at two different points (prior to the classroom activities and 12 months afterwards), to ascertain what improvement there had been in their knowledge and how sustained the effect of the educational activity undertaken by the teachers as multipliers (Fig. 3).

### Qualitative data collection and analysis

This approach determined what the teachers knew prior to the training courses and, after training, the teachers’ acceptance of the pedagogical strategies used in these courses.

Structured Interviews (SIs) and Focus Group Discussions (FGDs) were conducted face-to-face by FM and CM with a purposeful sample of 19 teachers, who participated in the training. The sample size was determined by the principle of theoretical saturation, where SIs and FGDs are carried out until a clear pattern appears and subsequent groups produce no new information [29, 30]. SIs were carried out and analysed before and after the training sessions to identify the teachers’ perceptions of schistosomiasis in the municipality and thus help construct the best pedagogical strategy for the training. After the educational activities conducted by the teachers with their students, FGDs were held in order to determine the teachers’ acceptance of the educational tools proposed during the training [31]. A semi-structured interview guide was used in the SIs and FGDs and was previously tested and validated in a smaller sample by the researchers. In the SIs, this script comprised open questions, 10 each on the etiology of the disease, classroom experiences, and memories, plus additional questions and instructions that allowed the interviewer to investigate in greater detail (Additional file 2). These questions were developed by the research team that works and has experience with schistosomiasis control and health education. The FGD questions addressed the educational processes experienced by the teachers with their students in the classroom after the training (Additional file 3).

The SIs and FGDs were carried out at school in a comfortable and quiet room where the interviewer, the observer and the participant were accommodated. The interviews lasted an average of 60 min, and the field notes were recorded by the interviewer and also by the observer. The SIs were performed twice with the same participant before and after the training courses.

Several themes emerged and were further explored during SIs and FGDs. Data collected by these methods helped clarify and interpret the results of the discussions. Individual accounts were translated into comprehensive concepts by textual analysis of the transcriptions.

The transcriptions of the interview and focus group recordings were input to the MAXQDA 10 programme. Qualitative analysis was performed using thematic content analysis and categories were generated after reading the transcripts. These categories were discussed to achieve an agreement between the researchers. Two researchers developed a codebook and performed line-by-line coding.

In developing the study design, the Consolidated Criteria for Reporting Qualitative Research (COREQ) were followed: a 32-item checklist for the SIs, and focus groups to ensure high-quality reporting. COREQ items help researchers report important aspects of the research team, the study methods and context, and the analysis and interpretation of results (Additional file 4).

The qualitative research team consisted of four specialised professionals, two men (FM, CM) and two women (LB, TF), all working in the health field and with experience in qualitative research and publishing. The study coordinator contacted the participants and, to avoid any undue influence, a different team member conducted the interview. The team members made every effort not to allow personal views and biases to influence data collection and analysis, and to prevent bias during drafting of the paper.

### Quantitative approach to pupil knowledge survey

This stage included all 276 students who received classes from the 19 teachers selected to participate in both courses.

In order to assess whether, after the course, the educational activities carried out by the teachers in the schools fixed knowledge about the disease, the pupils answered a structured questionnaire, validated in a previous study [10], (Additional file 5) on its aetiology, transmission, diagnosis, treatment, and related risk behaviour. This questionnaire was applied on two different occasions: before the activities and 1 year after the two courses, in order to ascertain whether there had been any change in their knowledge and whether the change endured for a year. Changes in responses before and after 1 year the educational activities were evaluated by the McNemar test. Percentages were calculated for qualitative variables, while medians and interquartile ranges (IQRs) were calculated for age. Stata 17.0 software was used. A map was drawn using QGIS 2.18.7 software geo-referencing the study schools.

### Parasitological survey and treatment

The infection status of teachers and students was assessed by a parasitological survey using the Kato-Katz quantitative method [32] using one stool sample on two slides. The selection of this method was to replicate the same diagnostic approach used by the health units of the municipality, even though this method has low sensitivity in areas of low schistosomiasis prevalence [33]. All teachers and pupils who tested positive for schistosomiasis and/or soil-transmitted helminthiasis were treated with praziquantel (a single dose of 600 mg/kg) and/or albendazole (a single dose of 400 mg), respectively, under the supervision of a local doctor.

## Results

### Parasitological test

In the parasitological study, six (31.6%) of the 19 teachers and 59 (21.4%) of the pupils participating in the study tested positive for *S. mansoni*.

### Teachers’ perceptions/knowledge of schistosomiasis before and after training

Table 1 below describes the four themes addressed and details the aspects that emerged after thematic content analysis of the SIs.

**Table 1:** Themes and aspects identified in the teachers’ interviews before and after training

Themes	Aspects
Aetiology of the disease	Signs and symptoms, accounts of clinical conditions, notions of diagnosis and treatment
The biological cycle of *Schistosoma mansoni*	Stages of the cycle, general notions about the intermediate host, risk behaviour, transmission sites, prevention and control measures
Context of the disease in the municipality	Perceptions of the endemic in the territory, identification of hazardous sites and the population affected, public policies
Schistosomiasis in the classroom	Descriptions of the teaching materials used, information sources and updating, importance of the subject in the classroom

#### Theme 1: Aetiology of the disease (before training)

On this theme, the teachers were found to have scientific knowledge of the disease, but it was fragmented. One teacher confused the disease with its agent; another failed to relate the disease with its agent of infection, but knew which organs were most affected by the disease; and a third identified the aetiological agent, but confused the concepts of host and parasite. On the other hand, nine teachers associated the disease correctly with its aetiological agent, *S. mansoni*.

“It is a disease caused by the host worm: *Schistosoma mansoni*” (Teacher 01).

As regards symptoms, nine participants mentioned diarrhoea, dizziness and tiredness and four teachers showed they knew the symptoms of neuroschistosomiasis. That observation agrees with the actual cases identified by the research team in the municipality.

*“At a very advanced stage, schistosomiasis can move to the spinal cord or to the brain”. (Teacher 02)*

In all the narratives, the meanings given to the disease could be seen to portray actual experiences, involving precise descriptions of signs and symptoms, either in family members or memories of a fairly recent past.

One teacher, in the prior SI, quoted a popular saying that mentions cercarial dermatitis.

*“...There’s the saying that if you went swimming and then started itching, it’s because you’ve caught it”.* (Teacher 3)

Proverbs are powerful everyday expressions of culture, which endure in society and change in meaning over the years; they grow out of popular knowledge on a subject and are loaded with symbolism. However, the popular saying the teacher referred to is at odds with scientific knowledge, because it suggests a false relation between cause and effect.

Two teachers did not know what laboratory test was used to diagnose the disease and two mentioned blood tests as the technique used to detect the parasite.

#### Theme 01: Aetiology of the disease (after training)

After the course, six teachers were able to distinguish the two main stages of the disease, the initial acute stage and the chronic stage. That concept is important because it makes it possible to understand that the patient can be treated early to prevent progression to the severe forms of the disease, thus mitigating the fear that the disease will lead to a fatal outcome.

After the course, five teachers related the disease to its parasite and one teacher mentioned the phylum (Platyhelminths) correctly, while another teacher mentioned the existence of two hosts. The distinction between disease and causative agent featured in all the teachers’ interviews, with additional meanings, as shown by the following quote.

*“It is a disease caused by a parasitic worm that has two hosts, one intermediate and the other definitive”. (Teacher 3)*

Three teachers associated the disease with pupils’ learning difficulties, demonstrating a new perception of the parasite’s impact on the pupils’ quality of life.

*“I saw that those pupils who did not feel like doing the activities in the classroom and outside were diagnosed with schistosomiasis”.* (Teacher 16)

On both occasions (before and after training), three teachers mentioned death as one outcome of the clinical complications arising from the disease.

*“The worm penetrates your organism through your skin or mouth and can give you disorders and even lead to death”.* (Teacher 14)

*“But when I was a child, someone my grandfather knew died and they said he had “water belly” (Ascites), because he looked pregnant”.* (Teacher 9)

The allusion to death reflects the memory of how severe schistosomiasis is in the region. The epidemiological situation revealed by the parasitological survey of schoolchildren in the municipality in 2013 indicated a prevalence of 21.4% [10].

After the course, all the teachers referred to stool testing as the surest method of diagnosing the disease. It was only after the course that three teachers made mention of treatment.

*“The best test to know whether you’ve got schistosomiasis is the stool test. Before, I thought it was the blood test”.* (Teacher 5)

#### Theme 02: Understanding the biological life cycle of *Schistosoma mansoni* (before training)

In the teachers’ interviews, a barrier was observed in their understanding of the parasite’s life cycle: some did not understand properly how transmission takes place in water and what role is played by the snail (the intermediate host).

*“…It can be transmitted when you go barefoot in contact with the contaminated ground”. (Teacher 13)*

Five teachers confused the manner of transmission of schistosomiasis and soil-transmitted helminthiasis:

*“Schistosomiasis is transmitted by contaminated faeces and that happens through the soil, water, and ingestion of contaminated food. The belly gets really infected, that is, big”. (Teacher 17)*

The accounts of 11 of the teachers mentioned the site of infection (water) correctly. One teacher said that infection occurs most effectively if the person has a wound before coming into contact with the water because the wound makes it easier for the parasite to penetrate.

*“You catch the disease when you come into contact with water in rivers and lakes, particularly when the person has a wound, because that makes it easier, doesn’t it?” (Teacher 07)*

Another teacher, probably associating information about dengue with schistosomiasis, said that infection occurs in clean, standing water.

*“The disease is contracted in places where there is clean, standing water…”. (Teacher 16)*

However, five teachers said that infection occurred only in water contaminated with human waste.

*“You catch schistosomiasis when you go into water contaminated with human faeces”. (Teacher 02)*

As regards the intermediate host snail, only four teachers mentioned that the disease was related to the presence of snails in the water. Of these four, one was succinct, but failed to relate the ideas he mentioned.

*“The disease is transmitted in water, snails, faeces”. (Teacher 14)*

As regards risk behaviour, only two teachers mentioned these explicitly, always framing them in terms of public health, but taking no account of the socio-environmental aspects of the disease.

*“People have to be made aware because otherwise you treat it and they are going to get infected again”. (Teacher 9)*

#### Theme 02: Understanding the biological life cycle of *Schistosoma mansoni* after training

The reference to human faeces as an environmental contaminant shows a degree of understanding of the human role in perpetuating the disease. In the post-training interviews, all the teachers mentioned faeces as an important, integral part of the parasite’s life cycle and the terms used by the teachers to refer to the place where infection takes place were predominantly “contaminated water” and “polluted water”.

During the SI after the course, all the teachers mentioned snails as an integral part of the disease cycle. Four teachers mentioned the genus name of the intermediate host snail (*Biomphalaria*) correctly.

*“There’s the snail (Biomphalaria) that lives in water and transmits cercaria…”. (Teacher 11)*

After the course, the discourse of eight of the teachers reflected greater concern with risk behaviour. However, these teachers’ discourse came to reveal greater concern with public social policies in controlling the disease in the municipality.

*“We have to demand sanitation for the population, because that is the only way we are going to put an end to schistosomiasis in Malacacheta”. (Teacher 19)*

*“We can only control the disease by the public administration’s being committed to the population (putting in sanitation, improving health care)”. (Teacher 13)*

After the course, it was noted that all the teachers understood that schistosomiasis is transmitted in bodies of water, which are where infection occurs. Seven teachers mentioned the occurrence of snails in aquatic environments as an important element in the cycle that maintains the endemic.

*“When a man comes into contact with water contaminated by cercaria, which developed before inside a mollusc (snail) (of the genus) Biomphalaria”. (Teacher 7)*

It is important to understand the parasite’s life cycle properly, because that makes it possible to understand the risk factors related to the disease and the transmission site, and allows the local population to understand prevention and control measures.

#### Theme 03: Context of the disease in the municipality before and after training

In the SIs before and after the training, all the teachers said they knew the disease existed in the municipality. Four teachers singled out the rural area as being the most affected by the disease.

*“There is schistosomiasis here, mainly in the rural area, because there’s more access to rivers and lakes”. (Teacher 5)*

These teachers’ perceptions are in line with other studies by the research team, which revealed higher prevalences in rural areas than in urban areas [34, 35].

Some teachers expressed negative perceptions of the medicine used in treatment, which date from the use of oxamniquine (Mansil®), a drug that causes a series of side effects, particularly on the nervous system, but which is no longer used in Brazil since in the 1990s.

*“The disease scared me, you know. The greatest fear was of the medicine, the symptoms it caused. They used to say Ah! You’ll go a bit mad”. (Teacher 12).*

After the course, three teachers’ discourse had absorbed concepts such as “endemicity” and had expanded their idea of the main sites of contamination in the municipality.

*“There’s a lot of schistosomiasis here in the region, because we live in an endemic area”. (Teacher 15)*

*“We have the disease most precisely in the districts which are more endemic areas, but there are cases in the main town too” (Teacher 01).*

It is important that, during the education process, the problem can be discussed on a territorial basis, along with the available epidemiological data. The maps that were assembled and problematised in this study constituted a powerful resource for generating discussion about the disease in the municipality.

There was a strong relationship with the disease in both the personal and family contexts, where the experience of contact with relatives and/or friends who had become ill was very present in the teachers’ memories.

*“Powerlessness is the most important word because I didn’t know what to do and we didn’t even know what it was – seeing my relatives in pain and having no remedy for the pain”.* (Teacher 19)

#### Theme 04: Schistosomiasis in the classroom before and after training

In the SIs before training, some teachers reported that schistosomiasis was responsible for a fair amount of school absenteeism. However, when asked whether they had even worked with the subject of schistosomiasis in the classroom, all the teachers declared that they had at least mentioned the disease. One teacher reported that she had addressed the subject only with upper secondary pupils. However, what that teacher said was observed to include mistaken information about the transmission of the disease, which was confused with soil-transmitted helminthiasis.

*“Yes, I have talked about schistosomiasis. I explained that they shouldn’t bathe in polluted rivers and should have hygienic habits like washing their hands well before meals and washing vegetables”. (Teacher 3)*

In the SI, it could be seen that the subject was rarely raised with the pupils and that the approaches were limited.

*“When I spoke about the subject, I was very brief”. (Teacher 6)*

On the other hand, in the SIs after the course, all the teachers talked about their plans for working on the subject with their pupils.

*“I never talked very clearly or openly, because my knowledge of schistosomiasis before did not go very deep”. (Teacher 8)*

### The teachers’ acceptance of the educational tools proposed during the training

During the two courses, it became clear that the teachers were committed to the health policies in the school and in the community. The experience and sharing among them and their use of pedagogical resources, such as drama, craft workshops, group dynamics and practical laboratory and fieldwork classes, were decisive to the success of the educational activities. One important point was that the process broke with the traditional model of course often offered to teachers. The teachers said that training of that kind is usually boring and lacking in innovation.

*“We have had other training courses, which were extremely tiring, and you can’t manage to absorb all the knowledge that way”.* (Teacher 01)

*“The course was really good. Today I can say that it completely changed the way we saw things: we used to teach it all wrong, but not today. If I could, I would do it again”. (Teacher 7)*

One teacher said that the major difficulty in working with the subject in activities in the schools was that they were overloaded with activities, because of a lack of support from other colleagues who were not involved in the course.

*“I did everything alone, because my colleague in the area or the subject left the school. That was why I was alone in giving the classes”. (Teacher 17)*

The teachers identified great interest in the subject among their pupils. The discussions revealed a proactive spirit among the teachers and a desire to bring change to the pupils’ lives.

*“We have to mobilise the public, we have to pressure the people in government to be more concerned with our health. We are teachers and we have that role”.* (Teacher 14)

### Pupil knowledge survey

More than half of the pupils evaluated before and after the educational activities lived in urban areas (58.69%), and 28.98% said they had already had the disease. The median age (IQR = 11.0–12.0) is 11 years, and 53.63% of them are boys.

Before the classroom activities, the pupils of teachers on both courses already showed a familiarity with schistosomiasis. Of those pupils, 224 (81.1%) knew someone who had had the disease, 29% reported having had the disease and 24.6% had heard reports of the disease at home.

The pupils’ responses to the questionnaire applied before the classroom activities also showed a satisfactory level of correct answers to questions requiring more specific knowledge about the transmission of the disease. Nonetheless, there was a significant (p < 0.05) difference in the pupils’ responses before and after the classroom activities conducted by the teachers, resulting from an increase in the percentage of correct answers. Correct answers to questions 1 to 4, before and after the educational activities, increased from 36.7% to 99% in Training Course I and from 75.8% to 97.8% in Training Course II (Table 2). Meanwhile, there was no significant difference in the high percentages of correct answers to question 5 (on contact with bodies of water) before and after the educational activities of both Training Course I (above 84%) and II (above 80%).

**Table 2:** Comparison of correct answers given by pupils of teachers who participated in Training I and II

Questions	Training Course I (n = 98)			Training Course II (n = 178)		
	Before n (%)	After n (%)	*p*	Before n (%)	After n (%)	*p*
1. How is schistosomiasis transmitted?	71/98 (72.4)	96/98 (98)	**0.0000**	152/178 (85.4)	173/178 (97.2)	**0.0000**
2. What animal transmits schistosomiasis?	36/98 (36.7)	96/98 (98)	**0.0000**	143/178 (80.3)	174/178 (97.8)	**0.0000**
3. Where does that animal live?	54/98 (55.1)	97/98 (99)	**0.0000**	135/178 (75.8)	173/178 (97.2)	**0.0000**
4. What test do you have to do?	51/98 (52.0)	92/98 (94)	**0.0000**	154/178 (86.5)	171/178 (96.1)	**0.0004**
5. Do you go into the water of rivers, streams? *	83/98 (84.7)	82/98 (83.7)	0.8273	143/178 (80.3)	136/178 (76.4)	0.2623

*In the question whether the pupils went into water for any reason, the response “Yes, I do” was considered correct, even though it represented risk behaviour

## Discussion

Stool tests revealed that high percentages of both the multiplier teachers (31.6%) and their pupils (21.4%) were positive for *S. mansoni*, while they were unaware of their infection status. That scenario contributed strongly to the teachers determined engagement in conducting the educational activities with their pupils, making them key components of the endeavour to control the disease. Several studies have suggested that teachers can facilitate and motivate pupils’ adherence to diagnosis and treatment, in addition to mobilising educational activities to develop a clearer understanding of the disease, thus making pupils and their families more willing to participate in activities led by teams of local health workers [16, 19, 36–39].

In order to leverage the teachers’ role and guarantee the success of the educational activities, it is fundamental that they understand the issue and are capacitated using approaches tailored to the epidemiological realities of the endemic area where they live and teach [18, 27]. Generic public health approaches do not meet the needs of local cultural practices and are less likely to be successful than those that do. Also, the latter, if intended to be sustainable, must encourage participation by the target community [40].

The two courses offered to the teachers in this study were intended precisely to conduct activities that would enable them to perceive and discuss the disease’s local context. This was done by way of practical laboratory activities and witnessing actual conditions in the field that favour transmission (Training Course I) and by participating in group play activities to enable them to identify and discuss the key factors involved in local transmission. In Training Course I, the methodology that was applied, culminating in a field visit, proved appropriate and motivating. The fact that they had learned of their infection status and identified the vector snails in situ and the main socio-environmental conditions in which the disease had become installed in the municipality may have contributed to their acquiring and fixing knowledge about the endemic, as well as committing to warning their pupils, colleagues, neighbours and the community and conveying that knowledge to them. In addition, the practical classes (diagnosis and observation of the stages of progression of *S. mansoni*) and the classes with videos and graphics made the course more pleasant and interesting, as was made clear in the focal group. In Training Course II, the activity of producing educational materials, including mock-ups and models, was what most roused the teachers’ interest, because it enabled them to step completely outside their usual role and become pupils again. The broader purpose of the play activities was to furnish materials and techniques that would help the teachers arouse the interest of their pupils, who had difficulty understanding the cycle of the disease, particularly those aspects that are not visible [41].

The education strategy adopted here resulted in a significant change in the teachers’ level of understanding of the disease and the epidemiological context in the municipality and substantial improvement in their pupils’ knowledge. The latter came to provide correct answers to questions denoting greater specificity, depth and complexity of the information involved. The discussion generated in the two focal groups showed that the knowledge that had been constructed and acquired by the teachers, and their motivation as multipliers of that knowledge persisted 1 year after the educational activities. The knowledge acquired by the pupils also continued significant (p = 0.0000, Table 2) after a year, demonstrating the sustainability and success of the activities conducted by the teachers. This corroborates the discussion of the power that transdisciplinary activities in schools have to spread health information [42, 43].

Despite the improvement in knowledge of the disease, most of the pupils continued in contact with bodies of water (Question 5), as observed in other studies [37, 39, 44]. One plausible explanation is that the municipality is located in a critical area where water resources are scarce and which has suffered from prolonged drought in recent years, as reflected in periodic lack of water in residences and schools. That climatic condition, added to the lack of leisure options, favours maintenance of this risk behaviour, which is also associated with hygiene and labour practices, particularly among schoolchildren living in rural areas [16].

The fact that the schoolchildren continued to enter the water does not invalidate the educational activities conducted by the teachers, because the knowledge the schoolchildren acquired can help them and their families to identify the presence of the intermediate host snail in bodies of water, to perceive signs and symptoms and associate them with contact with water, and then to alert health personnel and, consequently, facilitate diagnosis and treatment.

In Brazil, 96% of Brazilians from 6 to 17 years of age attend schools [45]. That high frequency is related to social mechanisms, such as income distribution programmes, which reduce absenteeism, because one of the criteria for the cash transfers is regular school attendance [46]. That unquestionably has educational benefits and opens up a range of possibilities for exploring health-related issues in that age range within the school environment, using teachers as promoters of knowledge [34]. For that purpose, teachers must be identified by local health teams as potential partners and be involved in capacity-building activities that equip them to play that role to the full.

In many endemic countries, schools have been used as bases for operating periodic mass drug administration (MDA) of praziquantel in schoolchildren [47]. That strategy has elicited the use of health education activities to foster greater awareness, preventive behaviour and adherence to the MDA cycle among schoolchildren. Many such programmes have failed to achieve the intended success [48], because social, cultural and environmental factors can interfere directly in the success of the approach [47, 49, 50]. On the other hand, Spencer et al. [40] successfully introduced a schistosomiasis education programme with community consultation, engagement and participation in an area of Madagascar, where prevalence of infection with *S. mansoni* was greater than 88% and the burden of disease among school-age children was high. At first, there were large gaps in knowledge about schistosomiasis and, after the programme, improvements were observed in understanding the disease, adherence to MDA and use of latrines. The participating schoolchildren’s adherence to MDA was 91% after the programme was introduced, more than twice the adherence among non-participating schoolchildren.

In Brazil, the Schistosomiasis Control Programme run by the Ministry of Health’s Health Surveillance Secretariat (*Programa de Controle da Esquistossomose da Secretaria de Vigilância em Saúde do Ministério da Saúde*, PCE/SVS/MS) recommends identifying carriers of infection, followed by treatment of those infected, in areas with prevalence of less than 25% (which is the situation in most of the endemic localities) and mass treatment only in those areas where prevalence is greater than 25% [51]. Accordingly, for the local PCE teams, the greatest bottleneck is the schoolchildren’s adherence to stool testing.

Here, therefore, it is fundamental that the activities conducted in schools encourage and sensitise the schoolchildren to adhere to the testing and to selective treatment [45] and to adopting preventive behaviour.

Several educational initiatives undertaken in schools or communities have been successful in improving school-age children’s knowledge of schistosomiasis, and have achieved greater adherence to diagnosis and treatment and fostered some changes in risky behaviour [16, 19, 37, 52]. Most, however, are ad hoc and external to the communities, and positive results are rarely sustained after termination of project funding [53]**.** This weakens the overall endeavour and the effectiveness of the expenditure involved and limits any evaluation of the long-term impact of health education measures in controlling the disease. It is no simple task to measure the impact of educational measures on a disease such as schistosomiasis, nor to guarantee that education programmes will yield effective change in the adoption of protective behaviour, although they are a vital tool in any programme to control neglected diseases [40].

For these reasons, it is fundamental to support endemic municipalities in creating permanent education programmes culturally suited to local realities, involving key actors, such as teachers, health personnel and community leaders who work to spread knowledge and continuously involve target groups.

It is widely known that, in order to attain the goal of eliminating schistosomiasis as a public health problem by 2030, it will be necessary to employ integrated multisector approaches that include treatment with praziquantel, access to safe water and sanitation and health education activities. These measures are known to have their intrinsic limitations determined by eco-epidemiological, socioeconomic, and cultural factors, which vary widely among endemic areas. Schistosomiasis is one of the neglected diseases that is strongly associated with factors that act to perpetuate poverty. Accordingly, it will cease to be a public health problem only when government measures reduce poverty, when conditions of life and housing become appropriate and when there is social and environmental justice.

## Conclusion

This study has offered suggestions and possible health education approaches in the context of schistosomiasis, with primary and lower secondary school teachers as its target group and as multipliers of knowledge among their pupils. As there were favourable changes in the teachers’ discourses from both training courses, together with improvements in their pupils’ knowledge, a future possibility of an educational strategy would be to combine the two approaches into a single capacity-building proposal. Also, the outcomes underline the importance of schools and teachers as indispensable partners for local health teams, as well as the need to develop sustainable, permanent education programmes tailored culturally to local realities, as part of government plans for combating schistosomiasis in endemic areas.

## Supplementary Information

**Additional file 1.** Content addressed, activities conducted and format of classes held with the teachers in the two training courses. https://doi.org/10.6084/m9.figshare.19990871.

**Additional file 2.** Semi-structured interview guide. https://doi.org/10.6084/m9.figshare.19990880.v1.

**Additional file 3.** Focus group discussion guide. https://doi.org/10.6084/m9.figshare.19990883.v1.

**Additional file 4**. Consolidated criteria for reporting qualitative research (COREQ). https://doi.org/10.6084/m9.figshare.19990865.v1.

**Additional file 5.** Schistosomiasis questionnaire applied to students. https://doi.org/10.6084/m9.figshare.20201246.v1.

## Data Availability

The data that support the findings of this study are available from Fundação Oswaldo Cruz, but their availability is restricted, as they were used under license for the current study, and so are not publicly available. Data are however available from the authors upon reasonable request and with permission from the Fundação Oswaldo Cruz.

## Abbreviations

WHO: World Health Organization
HDI: Human Development Index
SI: Structured interview
FGD: Focus Group Discussion
COREQ: Consolidated Criteria for Reporting Qualitative Research
IQR: Interquartile range
PCE: Schistosomiasis Control Programme
MDA: Mass drug administration
