# Supplementary material for: Teachers as multipliers of knowledge about schistosomiasis: a possible approach for health education programmes
Source: BMC Infect Dis. 2022 Nov 14;22:853. doi: 10.1186/s12879-022-07829-x (PMC9664691; doi:10.1186/s12879-022-07829-x)
Supplement: Supplementary file 1 — Additional file 1. Content addressed, activities conducted and format of classes held with the teachers in the two training courses. https://doi.org/10.6084/m9.figshare.19990871. [file 12879_2022_7829_MOESM1_ESM.pdf]

Supplementary Table S1. Themes, activities and format of the classes for teachers in the two study training courses

Themes	Activities	Class format
Training I – Creative-Pedagogical Approach		
Overall Aspects of Schistosomiasis	- Dialogue-based class on the transmission cycle, diagnosis, clinical aspects, epidemiology and control.<br>- Conversation circle: personal accounts and experiences shared among teachers and discussion of socio-environmental aspects of transmission of the endemic in the municipality.	Theoretical
The vector snail and diagnosis of *S. mansoni* infection	- Observation of *Biomphalaria spp.* snails and of other non-vector snails, plus the epidemiological importance of the species.<br>- Preparation of a malacological sample box of *Biomphalaria spp.* Snail shells for use with the pupils.<br>- Class on methods of diagnosing *S. mansoni* infection in the snails and observation of cercaria, the larva that infects humans.	Theoretical and practical (laboratory)

Diagnosing infection in humans	- Using the microscope to visualise the development stages of *S. mansoni* and of soil-borne helminths of medical importance to the municipality.<br>- Demonstration of the Kato-Katz technique for parasitological diagnosis of human infection.	Theoretical and practical (laboratory)
Pathology and treatment	- Video of dissection of infected and healthy mice, showing clinical forms of the disease (comparing size, form and colour of liver and spleen).<br>- Observation of adult worms conserved in formalin.<br>- Discussion of pathology, treatment and accounts of teachers' experiences with the disease.	Theoretical and practical (laboratory)
Health Education	- Presentation and discussion of the Health Education Strategies recommended in schools.<br>- Discussion prevention and control strategies implemented successfully in another endemic municipality in Minas Gerais.	Theoretical

Field Visit	- Visit to rural localities in the municipality to identify socio-environmental conditions that favour transmission and risk situations.<br>- Identification of natural habitats of *Biomplahalaria spp.* and collection of snails in breeding grounds.<br>- Examination of snails collected to detect natural infection with *S. mansoni.* (activity carried out by the researchers).	Theoretical and practical fieldwork
Workshop to build the "education kit"	- Collective construction by teachers and researchers of a kit of schistosomiasis-related materials and a guide for use in the educational activities with the pupils.	Theoretical and practical
Training II – Creative Play Approach		
Identification of schistosomiasis as a health problem	- Technique for building "Talking Maps", cognitive maps, used to encourage interaction with the teachers and development of maps portraying the collective representation of the territory where they live and the main health problems related to it.<br>- The technique of Schistosomiasis in images was used to facilitate learning by association	Theory and play

	with images that show the disease and reflect local realities.	
Biological concepts of schistosomiasis	- Practical class “Getting to know the vector snails and the parasite”, in three stages: (a) presentation of healthy specimens of *Biomphalaria*, (b) presentation of the malacological sample box of vector and non-vector snails found in the same bodies of water for purposes of comparison; and (c) examination of slides of fixed *S. mansoni* eggs, cercaria and adult worms to understand the stages of the parasite’s development.<br>- Creation workshop to develop teaching resources (models etc.) of the *S. mansoni* biological life cycle so as to fix knowledge and depict the conditions that favour transmission.	Theoretical and practical (laboratory)
Play techniques to evaluate and fix the content addressed	- **Knowledge Balloon Technique**<br>Group dynamic in which participants formulate questions on stimulus-theme (schistosomiasis) and place them inside a rubber balloon. These are thrown into the air for each participant to choose one and try to answer the questions it contains. The aim is to	Theory and play

	foster integration and discussion of doubts raised by the group on the theme.<br><br>- “The Health Education Theatre”, a show using drama techniques as a teaching resource and to guide construction of a play with schistosomiasis as the stimulus theme.<br><br>- Organisation of two groups of teachers to write (script, scenery and costumes) and stage plays on key elements of the endemic (snails, risk activities, diagnosis and control measures).	
	**- Stimulus technique using ambiguous scenarios**<br><br>Simulation of schistosomiasis- related situations based on the teachers accounts and mistaken or correct concepts addressed during the course, so that the teachers can take positions for or against them and explain their opinions.<br><br>- Staging of plays constructed by the teachers and completion of a protocol by the research team to evaluate whether aspects of the disease were addressed correctly.	Theory and play
