# Supplementary material for: Teachers as multipliers of knowledge about schistosomiasis: a possible approach for health education programmes
Source: BMC Infect Dis. 2022 Nov 14;22:853. doi: 10.1186/s12879-022-07829-x (PMC9664691; doi:10.1186/s12879-022-07829-x)
Supplement: Supplementary file 2 — Additional file 2. Semi-structured interview guide. https://doi.org/10.6084/m9.figshare.19990880.v1. [file 12879_2022_7829_MOESM2_ESM.pdf]

Semi-structured interview guide

Name: ____

Academic background: ______________________________________

School(s) where work(ed): ___________________________________

School years taught: _______________________________________

1. What are the most important diseases affecting people living in Malacacheta?
2. What is schistosomiasis?
3. How is it transmitted?
4. Is there schistosomiasis in your town? If so, what do you think are the areas where transmission occurs?
5. How can schistosomiasis be identified? How can someone diagnose the disease, that is, how do they know they are infected?
6. Do you know anyone who has schistosomiasis? Do you know what the disease causes in the person who is infected?
7. Have you talked about schistosomiasis in the classroom? If so, how? If not, why?
8. What year do you work on this subject with? Why?
9. How do you deal with the subject? What information sources do you use? If you do not discuss the subject or have difficulty doing so, explain the reasons.
10. How do you keep up to date on the subject?
