# Supplementary material for: Teachers as multipliers of knowledge about schistosomiasis: a possible approach for health education programmes
Source: BMC Infect Dis. 2022 Nov 14;22:853. doi: 10.1186/s12879-022-07829-x (PMC9664691; doi:10.1186/s12879-022-07829-x)
Supplement: Supplementary file 3 — Additional file 3. Focus group discussion guide. https://doi.org/10.6084/m9.figshare.19990883.v1. [file 12879_2022_7829_MOESM3_ESM.pdf]

Focus Group Discussion Guide

Date: ____/____/____

Moderator: ______________________________

Observers: ____________________________

Participants: _____________________________

- Introduction of participants
- Introduction of moderator
- Introduction of observer

1. What did you think of the course on schistosomiasis that was given by our group?
2. Did you think the number of hours was enough?
3. What can you say about the theoretical content? And the practical content?
4. What was the most interesting moment in the course?
5. What was the least interesting moment?
6. Do you think it is important to know more about this disease? Why?
7. Before the course, did you know that schistosomiasis was transmitted by a snail?
8. Now do you know how to recognise that snail?
9. Where does it live?
10. Had you seen this snail anywhere in the town?
11. Were you curious to see whether there are vector snails near where you live?
12. What are the activities where you run the risk of catching schistosomiasis?
13. Does anyone in your family engage in schistosomiasis-related risk activities?
14. How was schistosomiasis approached in the classroom?
15. What was the greatest difficulty in covering the subject?
16. Which activity with the pupils gave you most pleasure?
17. What was the pupils’ participation like?

18. Did you notice whether they were interested? Was it a stimulating subject?

19. What did the pupils think of the material you offered them?

20. Was that material sufficient?

21. Did the pupils produce any kind of material that was presented in the classroom?

22. Do you feel motivated to work on this subject next year with other groups of pupils?
