# Supplementary material for: Teachers as multipliers of knowledge about schistosomiasis: a possible approach for health education programmes
Source: BMC Infect Dis. 2022 Nov 14;22:853. doi: 10.1186/s12879-022-07829-x (PMC9664691; doi:10.1186/s12879-022-07829-x)
Supplement: Supplementary file 4 — Additional file 4. Consolidated criteria for reporting qualitative research (COREQ). https://doi.org/10.6084/m9.figshare.19990865.v1. [file 12879_2022_7829_MOESM4_ESM.pdf]

Consolidated criteria for reporting qualitative studies (COREQ): 32-item checklist

Developed from:
Tong A, Sainsbury P, Craig J. Consolidated criteria for reporting qualitative research (COREQ): a 32-item checklist for interviews and focus groups. *International Journal for Quality in Health Care*. 2007. Volume 19, Number 6: pp. 349 – 357

No. Item	Guide questions/description	Reported on Page #
**Domain 1: Research team and reflexivity**		
Personal Characteristics		
1. Inter viewer/facilitator	*Which author/s conducted the interview or focus group?*<br><br>FM, CM	Qualitative data collection and analysis, 7.
2. Credentials	*What were the researchers' credentials?*<br><br>PhD, MD, BSc, MSc, MRes,	Qualitative data collection and analysis, 8.
3. Occupation	*What was their occupation at the time of the study?*<br><br>Physicians, university professors, and qualitative researchers.	Title page.
4. Gender	*Was the researcher male or female?*<br><br>Some researchers were male, others were female.	Qualitative data collection and analysis, 8.
5. Experience and training	*What experience or training did the researcher have?*<br><br>Renowned researchers, as well as graduate students with experience in qualitative research, all with qualitative research training and with published articles in the area.	Qualitative data collection and analysis, 8.
Relationship with participants		
6. Relationship established	*Was a relationship established prior to study commencement?*<br><br>None of the participants had an established relationship with an author prior to study commencement.	Study design and participants, 6.
7. Participant knowledge of the interviewer	*What did the participants know about the researcher? (e.g., personal goals, reasons for doing the research).*<br><br>Participants were briefed on the purpose of	Ethical considerations, 26.

	the study and understood that it was a research project for FIOCRUZ. Educational ethical approval had been granted and participants reviewed the participant information documentation prior to giving their written informed consent to be involved.	
8. Interviewer characteristics	*What characteristics were reported about the inter viewer/facilitator? e.g., bias, assumptions, reasons and interests in the research topic.*<br><br>No interviewer-related biases were identified.	Qualitative data collection and analysis, 9.
**Domain 2: study design**		
Theoretical framework		
9. Methodological orientation and Theory	*What methodological orientation was stated to underpin the study? e.g., grounded theory, discourse analysis, ethnography, phenomenology, content analysis.*<br><br>Inductive and deductive coding with thematic content analysis.	Qualitative data collection and analysis, 8.
Participant selection		
10. Sampling	*How were participants selected? e.g., purposive, convenience, consecutive, snowball.*<br><br>Intentionally, purposeful sample.	Qualitative data collection and analysis, 7.
11. Method of approach	*How were participants approached? e.g., face-to-face, telephone, mail, email.*<br><br>We did a face-to-face interaction with the teachers.	Qualitative data collection and analysis, 7.
12. Sample size	*How many participants were in the study?*<br><br>19 teachers	Qualitative data collection and analysis, 7.
13. Non-participation	*How many people refused to participate or dropped out? Reasons?*<br><br>No one has given up on participating in the project.	N/A
Setting		
14. Setting of data collection	*Where was the data collected? e.g., home, clinic, workplace.*<br><br>The SIs and FGDs were carried out at school	Qualitative data collection and analysis, 8.

15. Presence of non-participants	*Was anyone else present besides the participants and researchers?*<br><br>No, only the interviewer, the observer and the participant were in the room during the interview.	Qualitative data collection and analysis, 8.
16. Description of sample	*What are the important characteristics of the sample? e.g. demographic data, date*<br><br>The study contemplated 19 primary and lower secondary school teachers, five of them men and 14 women. Their backgrounds were multidisciplinary, length of teaching experience ranging from four to 15 years and they taught at the study schools.	Study design and participants, 6.
Data collection		
17. Interview guide	*Were questions, prompts, guides provided by the authors? Was it pilot tested?*<br><br>The questions were developed by Qualitative Research Team (QRT) and previously tested and validated by the researchers in a smaller sample of volunteers and adjustments were made to ensure the transparency and relevance of the questions.	Qualitative data collection and analysis, 8.
18. Repeat interviews	*Were repeat inter views carried out? If yes, how many?*<br><br>Yes, the SIs were performed twice with the same participant before and after the training courses.	Qualitative data collection and analysis, 8.
19. Audio/visual recording	*Did the research use audio or visual recording to collect the data?*<br><br>The interviews were recorded and transcribed without personal identifiers, so that the database could be anonymized	Qualitative data collection and analysis, 8.
20. Field notes	*Were field notes made during and/or after the inter view or focus group?*<br><br>Field notes were recorded by the interviewer and also by the observer.	Qualitative data collection and analysis, 8.
21. Duration	*What was the duration of the interviews or focus group?*<br><br>The interviews lasted an average of 60 minutes	Qualitative data collection and analysis, 8.

22. Data saturation	*Was data saturation discussed?*<br><br>The number of interviews was determined by the principle of theoretical saturation where SIs are carried out until a clear pattern appears and subsequent groups do not produce new information	Qualitative data collection and analysis, 7.
23. Transcripts returned	*Were transcripts returned to participants for comment and/or correction?*<br><br>No.	N/A
**Domain 3: analysis and findings**		
Data analysis		
24. Number of data coders	*How many data coders coded the data?*<br><br>Two researchers independently developed a codebook and performed line-by-line coding.	Qualitative data collection and analysis, 8.
25. Description of the coding tree	*Did authors provide a description of the coding tree?*<br><br>No.	N/A
26. Derivation of themes	*Were themes identified in advance or derived from the data?*<br><br>The analysis of the interviews and the field notes allowed us to identify the four major themes.	Qualitative data collection and analysis, 8.
27. Software	*What software, if applicable, was used to manage the data?*<br><br>The recordings of the Sis and FDGs were transcribed and inserted in the MAXQDA 20 program	Qualitative data collection and analysis, 8.
28. Participant checking	*Did participants provide feedback on the findings?*<br><br>No.	N/A
Reporting		
29. Quotations presented	*Were participant quotations presented to illustrate the themes/findings? Was each quotation identified? e.g. participant number*<br><br>Yes, quotations were presented to illustrate the themes/findings, and each quotation was identified with a participant number.	Results, 11-19.
30. Data and findings consistent	*Was there consistency between the data presented and the findings?*	Discussion, 20-25.

	Yes, there was consistency between the data presented and the findings.	
31. Clarity of major themes	*Were major themes clearly presented in the findings?*<br><br>Yes, major themes were clearly presented in the Results section using specific sections regarding each theme.	Results, 11-19.
32. Clarity of minor themes	*Is there a description of diverse cases or discussion of minor themes?*<br><br>No, minor themes were not discussed.	N/A
