# Supplementary material for: Teachers as multipliers of knowledge about schistosomiasis: a possible approach for health education programmes
Source: BMC Infect Dis. 2022 Nov 14;22:853. doi: 10.1186/s12879-022-07829-x (PMC9664691; doi:10.1186/s12879-022-07829-x)
Supplement: Supplementary file 5 — Additional file 5. Schistosomiasis questionnaire applied to students. https://doi.org/10.6084/m9.figshare.20201246.v1. [file 12879_2022_7829_MOESM5_ESM.pdf]

Questionnaire applied to students on knowledge about schistosomiasis

( ) BEFORE EDUCATIONAL ACTIVITIES Date: ________________

( ) AFTER EDUCATIONAL ACTIVITIES Date: ________________

Please give some information on what you know about the disease called schistosomiasis.

1. Name: ____________________________________

2. Study ID: ________ (completed by the team)

3. School: _______________

4. Year/Class: ______________________

5. Age: ________

6. Sex: ( ) Female ( ) Male

Have you ever heard of schistosomiasis?

( ) Yes, at home ( ) Yes, at school ( ) Yes, on television ( ) Yes, at the Primary Care unit ( ) Yes, in a teaching text ( ) Yes, talking to friends ( ) No, I have never heard of it

Has this subject been talked about at your school?

( ) Yes, the teacher talked about it ( ) Yes, a classmate talked about it ( ) Yes, it w as talked about at the school fair ( ) No, it has not been talked about

1. How is schistosomiasis transmitted, that is, how does someone catch this disease?

( ) in water ( ) in soil ( ) though the air ( ) I do not know

2. What animal transmits schistosomiasis?

( ) mosquito ( ) kissing beetle ( ) snail ( ) I do not know

3. Where does that animal live?

( ) in the air - flying ( ) on the ground - crawling/walking ( ) in water ( ) I do not know

4. How do you know if you have schistosomiasis; what test do you have to do to find out?

( ) blood test ( ) stool test ( ) urine test ( ) There is no test ( ) I do not know

5. Can schistosomiasis be treated?

( ) Yes ( ) No

6. Do you go into these rivers, streams or lakes?

( ) Yes ( ) No
